# Pregabalin and hyperbaric oxygen therapy on pain thresholds and anxio-depressive behaviors in a preclinical fibromyalgia pain model

**DOI:** 10.3389/fpain.2023.1097457

**Published:** 2023-03-01

**Authors:** Cassie M. Argenbright, Michelle K. Bland, Sydney L. Michener, Judy R. Wilson, Perry N. Fuchs

**Affiliations:** ^1^ Department of Psychology, The University of Texas at Arlington, Arlington, TX, United States; ^2^ Department of Kinesiology, The University of Texas at Arlington, Arlington, TX, United States

**Keywords:** fibromyalgia, acidic saline, hyperbaric oxygen therapy, pregabalin, anhedonia, anxiety

## Abstract

Fibromyalgia (FM) is a chronic, widespread pain disorder generally of a non-inflammatory nature with many known affective and cognitive comorbidities. There is promise in the implementation of hyperbaric oxygen therapy (HBO_2_) for alleviating FM pain and comorbidities, despite no work investigating the efficacy of this treatment in prominent preclinical FM models. This project aimed to investigate the affective components, specifically anhedonia and anxiety, associated with an acidic saline model of FM in rats. We investigated the acidic saline model's ability to produce the sensory component of FM through reduced mechanical thresholds, as well as anxiety-like and avoidance behaviors through measures of open field and place escape/avoidance. We further investigated the use of pregabalin, a known FM therapeutic agent, in reducing negative sensory and affective measures within the model. Results revealed insignificant between-group differences for measures of anxiety, despite animals in the FM condition showing significantly reduced mechanical thresholds. Results further revealed that the acidic saline model was effective in increasing place escape/avoidance behavior among animals in the FM condition, with pregabalin reducing avoidance behaviors. In addition, we investigated the role of HBO_2_ [two 60-minute treatments at 2.0 ATA (atmospheres absolute)] in alleviating FM-like pain, anxiety, and anhedonia in the acidic saline model, utilizing mechanical paw withdrawal thresholds, open field, and sucrose preference measures. Results revealed that the acidic saline model produced reduced thresholds indicative of FM-like pain. Data did not provide support for the presence of anxio-depressive comorbidities associated with the FM model. HBO_2_ treatment did not significantly increase mechanical thresholds as expected. Future studies should seek to investigate the experimental circumstances within which the acidic saline model produces negative affect alongside hyperalgesia in order to contribute to the development of a multidimensional FM treatment methodology.

## 1. Introduction

Chronic widespread pain disorders, such as fibromyalgia (FM), are highly represented in our population but well understudied. Upwards of 2% of the U.S. population suffers from FM, with women being two-fold more likely to be diagnosed (1). The lack of clinical understandings of the disorder and largely ineffective treatment modalities on a multidimensional level has resulted in a large fiscal and societal burden (2). The current 2016 American College of Rheumatology diagnostic criteria for FM in adults includes: (1) generalized pain in at least 4 of 5 regions; (2) symptom presence for at least 3 months; (3) a Widespread Pain Index (WPI) score ≥7 and a Symptom Severity (SS) score ≥5, *or* a WPI score of 4–6 and a SS score ≥9; and (4) FM diagnosis does not exclude the presence of other clinical disorders (3). The current diagnostic criteria have allowed for FM to be assessed along a continuum of its unique symptoms, providing a more precise quantification of widespread pain while reducing misclassification or discrimination based on the presence of coexisting disorders (3). The inclusion of more clearly defined FM diagnostic criteria on a multidimensional level has proven vital in that FM is often both exacerbated and defined by its affective and cognitive dimensions—especially in the development of preclinical representations of the disorder—including depression and anxiety, difficulty sleeping, and various cognitive deficits (4–6). The close relationship between FM and its association with affective disturbances demonstrates the need for diagnostic methods to be inclusive of the negative emotionality associated with the disorder.

Without a definitive etiological understanding of FM, it has been difficult to develop a preclinical model of FM that allows for the comprehensive study of negative emotionality associated with this pain disorder (7). While there are a handful of models developed for preclinical FM research, we focus on Sluka et al.'s acidic saline model due to long-lasting incorporation of both central and peripheral pain factors with the absence of peripheral tissue damage which is commonly found in FM patients (8). The model utilizes repeated injections of 4.0 ± 0.1 pH saline into the left gastrocnemius muscle of the rodent to induce a bilateral hyperalgesia lasting up to 4 weeks (8). Previous research has focused on the ability of the acidic saline model to induce a comprehensive FM-like experience, including the decreased pain thresholds and anxio-depressive characteristics of the disorder. Some studies have reported that the acidic saline model induces both anxiety- and depression-like behaviors (9–15). However, Pratt et al. provide evidence that the acidic saline model may not be as efficacious in the replication of FM-related affective behaviors as previously thought (16). While it is sufficiently understood that acid-induced hyperalgesia adequately models the sensory and possible etiology of FM, the conflicting results on the model's ability to induce negative emotionality, such as anxio-depressive symptoms, calls for further investigation into potential alterations of emotionality associated with the model.

In the study of chronic pain, hyperbaric oxygen (HBO_2)_ has provided evidence of anti-nociceptive and anti-inflammatory mechanisms (17), with efficacy of treatment having been shown in animal models of injury-related pain (18), carrageenan-induced paw edema (19), arthritic pain (20), and neuropathic pain (21). Within the clinical literature, a randomized controlled trial conducted by Yildiz et al. indicates that patients diagnosed with FM experienced a significant decrease in FM-associated tender points and visual analog scale scores, alongside an increase in pain thresholds (22). Efrati et al. also investigated the potential role of HBO_2_ in a clinical FM population and also found that treatment was effective in increasing physical pain thresholds and decreasing tender point count (23). Additionally, the same study found a significant increase in physical functionality as measured by the Fibromyalgia Impact Questionnaire (FIQ), a decrease in psychological distress as measured by the Symptom Check List (SCL-90), and an overall increase in quality of life (QoL) assessments (23). A more recent randomized controlled trial by Hadanny et al. showed improvement in women's scores on various measures of FM pain, such as the WPI, alongside improvement in various measures of PTSD (24). Further study, by Curtis et al. sought to validate the ability of HBO_2_ to both improve and maintain physical and affective FM symptoms (25). Curtis et al. also reported similar results as seen in other randomized controlled trials, with functional impairment symptoms, anxio-depressive symptoms, and sleep disturbances all being alleviated for 3 months following treatment (25). The use of HBO_2_ for the attenuation of both negative physicality and emotionality, such as depression- and anxiety-like behaviors, among various types of pain models and central nervous system disorders has persisted through both clinical (17, 26–28) and preclinical studies (17, 29). With FM being a disorder strongly associated with negative affective components, there is promise in the potential therapeutic benefits of hyperbaric treatment for both alleviation of FM-related hyperalgesia, as well as its related affective dimensions.

With much of our knowledge of the clinical management and underlying etiology of FM stemming from information obtained from preclinical models, it is crucial to understand to what extent these animal models fully mirror the disorder and to what extent these models can be utilized for further experimental treatment studies. Therefore, we examined these goals in relation to the acidic saline model's ability to replicate alterations of emotionality by measuring anxio-depressive like behaviors, while further investigating the efficacy of a known FM therapeutic agent, pregabalin, in alleviating sensory and affective FM symptoms. In addition, we further investigated the validity of the acidic saline model in producing anxio-depressive FM symptoms, as well as explored the role of HBO_2_ as a therapeutic approach for treating alterations of the sensory and affective dimensions of symptoms associated with FM.

## 2. Materials and methods

### 2.1. Subjects

Ninety-six female Sprague Dawley rats purchased from Charles River (225–250 g) were singly housed with access to food and water *ad libitum*. All animals were maintained on a 12-hour light/dark cycle. All procedures for this study were approved by the University of Texas at Arlington Institutional Animal Care and Use Committee (IACUC).

### 2.2. Mechanical paw withdrawal threshold (MPWT)

To quantify mechanical hypersensitivity associated with the acidic saline model of FM and the efficacy of pregabalin and HBO_2_ treatment, animals were placed into a Plexiglass chamber with a wire mesh bottom that allowed access to the plantar surface of the hind paw and habituated for 10 min. Mechanical sensitivity utilized von Frey monofilaments (3.85, 5.68, 9.74, 18.39, 39.42, 77.3, 135.3, and 251.34 mN) and quantified using the up-down method (30). Each trial began with the 9.74 mN filament delivered to the left hind paw for approximately 1 s, then similarly to the right hind paw. If no withdrawal response was observed (i.e., licking or paw withdrawal), the next highest filament force was used. If a withdrawal response was observed, the next lowest force was used. This procedure was repeated until there was no response from the animal at the highest force (251.34 mN) or until a total of 5 stimuli were administered. The 50% paw withdrawal threshold for each trial was calculated using the following formula: [Xth]log = [vFr]log + ky, where [vFr] is the force of the last von Frey used, *k* = 0.2593 is the average interval (in log units) between the von Frey monofilaments, and y is a value that depends upon the pattern of withdrawal responses. If an animal did not respond to the highest von Frey monofilament (251.34 mN), then *y* = 1.00 and the 50% mechanical paw withdrawal response for that paw was calculated to be 456.63 mN. This test was conducted 3 times, with the scores from each trial being averaged to determine the mean withdrawal threshold for the left and right hind paw of the animal. A combined mechanical threshold score was then averaged from the right and left paw values for each MPWT test for statistical analysis.

### 2.3. Open field test (OFT)

The open field test was used to measure motor activity as an indication of anxiety-like behavior in FM (or control) animals that were treated with pregabalin or HBO_2_. Animals were placed in the center of a circular open field chamber with a wooden base and aluminum sheet metal walls. A video tracking system (Ethovision) was used to record and quantify total distance traveled, total distance within the center of the chamber, and total distance traveled along the perimeter during a 5 min test period. Time spent along the perimeter of the circular chamber is an indication of higher levels of anxiety, whereas time spent in the center region of the chamber indicates less anxiety. For half the animals, the number of rears during the 5-minute time span was also recorded to further assess changes in exploratory behavior.

### 2.4. Place escape/avoidance paradigm (PEAP)

To quantify the affective component of pain in animals that were treated with pregabalin, we utilized a modified version of the escape/avoidance paradigm (PEAP) (31). Animals were placed in a half-black/half-white Plexiglass chamber with a wire mesh bottom to allow access to the plantar surface of the hind paws. Traditionally, the PEAP involves a unilateral pain model (i.e., L5 ligation of either the left or right hindpaw) and mechanical stimulation is applied every 15 s across a 30 min test period. Mechanical stimulation is applied to the “injured” paw when the animal is within the preferred dark side of the chamber and the non-injured paw when the animal is within the light side of the chamber (32). Since the acidic saline model produces a bilateral hyperalgesia, we modified the methodology such that von Frey stimulation (476 mN) to the hindpaws was alternated (left vs. right) each time while the animal was on the dark side of the chamber, whereas no mechanical stimulation was applied while the animal was within the light side of the chamber.

### 2.5. Sucrose preference test

The sucrose preference test was used as a measure of anhedonia in animals that were treated with HBO_2_. Two bottles were available to the animals *ad libitum* for 72 h while within their home cage—one bottle contained a sweetened sucrose solution (2% wt/vol concentration) and the other bottle contained standard reverse osmosis water. The location (right side or left side of the cage) of the bottles was switched every 24 h to control for possible bias due to a place preference. The 2% wt/vol sucrose solution was made by dissolving 10 g of sucrose into 500 ml of reverse osmosis water. Volume measures (ml) of consumption from each bottle was taken every 24 h following the second isotonic saline or control treatment. Sucrose preference was calculated as: (% preference = [total sucrose intake/total fluid intake] × 100).

### 2.6. Procedures

Half of the animals were subjected to baseline measures of MPWT and OFT before being randomized to a pain condition (4.0 pH saline—FM; *n* = 48) vs. vehicle control (C; *n* = 48). For the induction of the acidic saline model of musculoskeletal pain, animals received a 4.0 ± 0.1 pH saline injection into the left gastrocnemius muscle while under anesthesia (isoflurane, 3% induction and 2% maintenance) whereas the saline control group received an injection of sterile saline. After five days, MPWTs were measured to ensure there was no hypersensitivity in either hind paw immediately prior to the second injection of acidic saline or normal saline. 24-hours after the second gastrocnemius injection, animals underwent MPWT and OFT tests to measure changes in post-induction withdrawal thresholds and anxiety-like behaviors. Animals were then randomized to a treatment condition (30 mg/kg i.p. pregabalin (PG) (Sigma-Aldrich, St. Louis, MO) vs. saline control (C)). One hour after treatment, subsequent measures of MPWT, PEAP, and OFT were collected. Final group randomizations were as follows: FM/PG (*n* = 12), FM/C (*n* = 12), C/PG (*n* = 12), and C/C (*n* = 12).

The other half of the animals (*n* = 24 FM; *n* = 24 saline) were separately randomized to receive hyperbaric oxygen therapy (HBO_2_) treatment or a control treatment (C). Animals randomly assigned to the HBO_2_ treatment condition were subjected to 1 h of 2.0 ATA. Animals assigned to the control treatment condition were placed in the chamber and experienced no manipulation of oxygen or pressure over the 1-hour period. 24-hours later, animals underwent their first post-treatment MPWT immediately before beginning their second hyperbaric or control treatment session. Upon completion of the second HBO_2_ treatment session, sucrose and water consumption was measured during three 24-hour periods (total of 72 h). 24-hours following the second HBO_2_ treatment session, animals were subjected to a 5-minute OFT to measure anxiety-like behaviors. Alongside daily measurements of sucrose preference, animals underwent MPWT tests every 24 h over the 72-hour period following the second treatment session. Measuring mechanical thresholds and anhedonic behaviors for 3 days following the second hyperbaric (or control treatment) session allowed for the detection of both acute and/or persistent therapeutic effects. Final group randomizations were as follows: FM/HBO_2_ (*n* = 12), FM/C (*n* = 12), C/HBO_2_ (*n* = 12), and C/C (*n* = 12).

### 2.7. Statistical analysis

The data were analyzed using SPSS (IBM SPSS Statistics 28). A one-way analysis of variance (ANOVA) was used to analyze the mean mechanical thresholds for pain condition (acidic saline or normal saline). To analyze the effects of pregabalin treatment, a one-way ANOVA was used to assess mean mechanical thresholds among groups (FM/PG, FM/C, C/PG, C/C). An additional mixed-model analysis of variance (ANOVA) was used to evaluate mean hind paw pain thresholds among groups treated with HBO_2_ (FM/HBO_2_, FM/C, C/HBO_2_, C/C) and across time (baseline, pre-treatment, post-treatment, 24-hours post-treatment, 48-hours post-treatment, 72-hours post-treatment). A mixed-model ANOVA was used to analyze changes in open field activity (total distance traveled and rearing behavior) among groups treated with pregabalin (FM/PG, FM/C, C/PG, C/C) or HBO_2_ (FM/HBO_2_, FM/C, C/HBO_2_, C/C) and across time (baseline, pre-treatment, post-treatment). A mixed-model ANOVA was used to assess avoidance behavior in the PEAP paradigm among groups (FM/PG, FM/C, C/PG, C/C) and across time (across 30 min in 5-minute time bins). A mixed-model ANOVA was used to analyze differences in sucrose preference among groups treated with HBO_2_ (FM/HBO_2_, FM/C, C/HBO_2_, C/C) and across time (24-hours post-treatment, 48-hours post-treatment, 72-hours post-treatment). All *post hoc* analyses were performed using Fisher's LSD (least significant difference) to control for type 1 error rate.

## 3. Results

### 3.1. Mechanical thresholds

**Figure 1:**
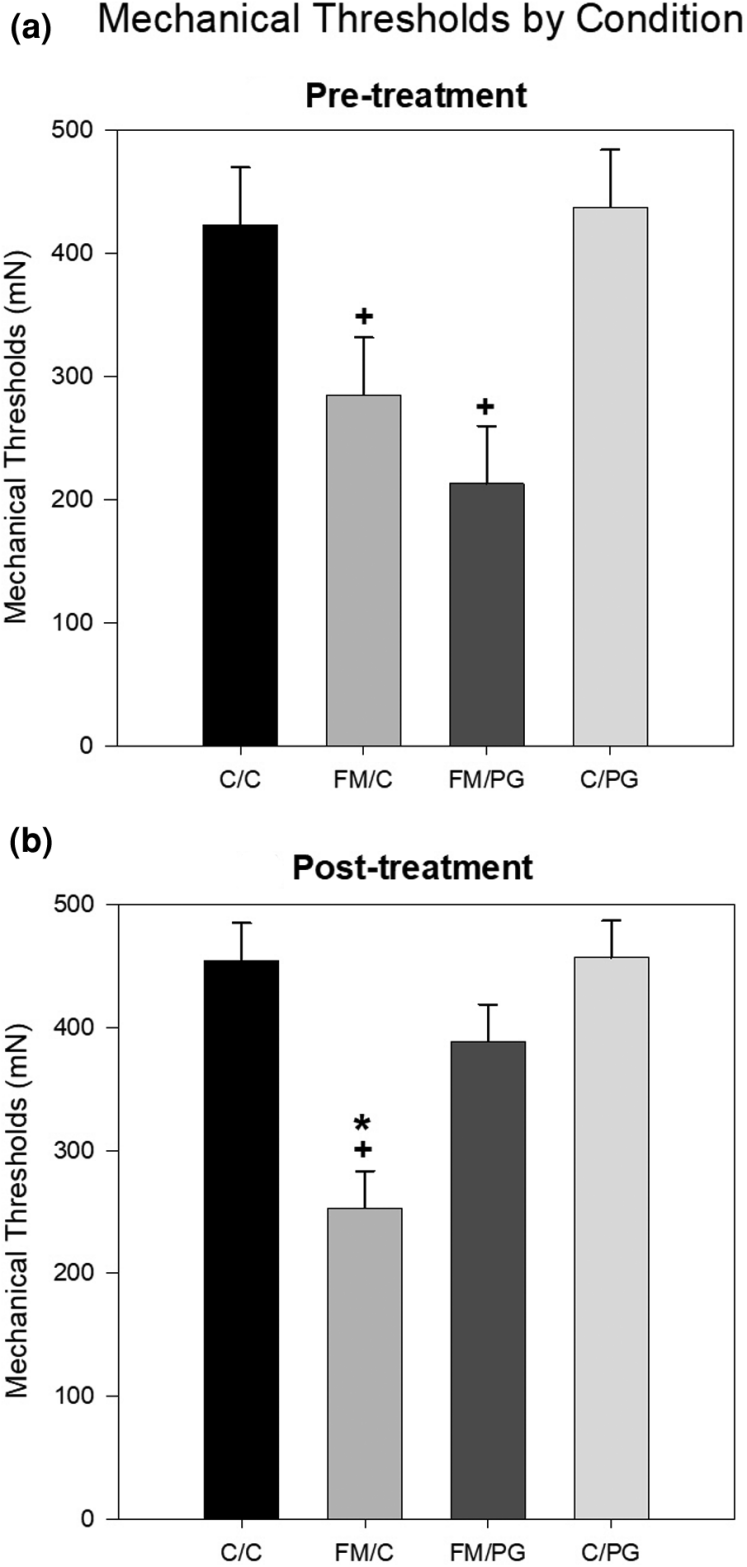
Mean mechanical thresholds were analyzed at pre-treatment and post-treatment with a one-way ANOVA. (**A**) Pre-treatment mechanical paw withdrawal thresholds (MPWT) by pain conditions displayed as mean (±SEM). (**B**) Post-treatment MPWT by pain conditions displayed as mean (±SEM). +*p* < .001 compared to C/C and C/PG groups, *p < .001 compared to FM/PG group.

*Pregabalin*: To analyze between-group differences in hyperalgesia prior to pregabalin or control treatment, a one-way analysis of variance (ANOVA) revealed a significant difference among groups, *F*(3, 48) = 10.89, *p *< .001. Animals randomized to the FM condition showed significantly reduced thresholds (i.e., hypersensitivity) compared to animals in the control condition (Figure 1A). Following pregabalin (or vehicle) treatment, a one-way ANOVA revealed a significant difference among groups, *F*(3, 48) = 20.30, *p *< .001. Animals in the FM/PG condition showed an increase in mechanical thresholds (less hypersensitivity) compared to vehicle treated controls, whereas animals in the FM/C condition continued to show mechanical hypersensitivity (Figure 1B).

**Figure 2:**
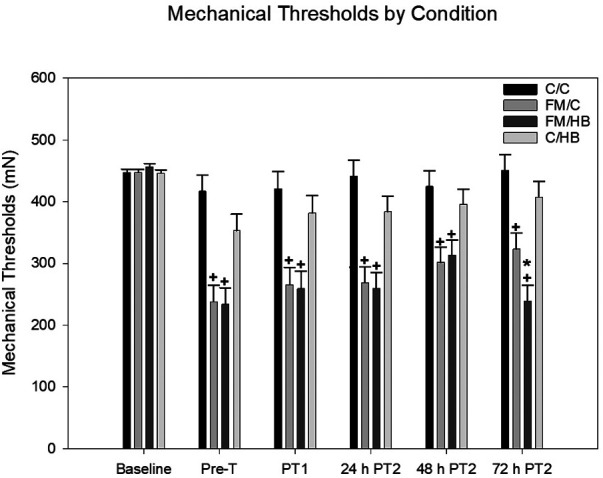
Mixed-model ANOVA of mechanical paw withdrawal thresholds (MPWT) by pain conditions over time displayed as mean (±SEM). Pre-T = Pre-treatment, PT = Post-treatment. + *p* < .05 compared to C/C and C/HB groups, *p < .05 compared to FM/C group.

*HBO_2_*: To analyze changes in mechanical thresholds of animals over time, a 4 (group) × 7 (time) mixed-model ANOVA was used, with group as the between-subjects variable and time as the within-subjects variable. A significant main effect for group, *F*(3, 44) = 17.517, *p* < .001, and time, *F*(5, 220) = 24.894, *p* < .001, was found. A significant group × time interaction was also revealed, *F*(15, 220) = 4.061, *p* < .001. *Post hoc* analyses revealed that, overall, animals in the acidic saline condition had significantly lower mechanical thresholds than saline controls. In terms of time, baseline mechanical thresholds prior to the induction of FM were significantly higher than thresholds at all other time points. Following two injections of acidic saline, FM condition animals had significantly lower thresholds than saline controls. There was no difference in thresholds seen in control animals over time, regardless of whether they received HBO_2_ or a control treatment. After each treatment, mechanical thresholds of animals in the FM/HBO_2_ group were not significantly different compared to animals in the FM/C group. This was contrary to our expectations, as we hypothesized that mechanical thresholds for animals in the FM/HBO_2_ group would increase significantly after each treatment session. At 48 h following the second treatment, no differences were observed between FM/HBO_2_ and FM/C groups. However, 72 h following the second treatment, the FM/HBO_2_ group showed significantly reduced thresholds compared to the FM/C group (Figure 2).

### 3.2. Open field

**Figure 3:**
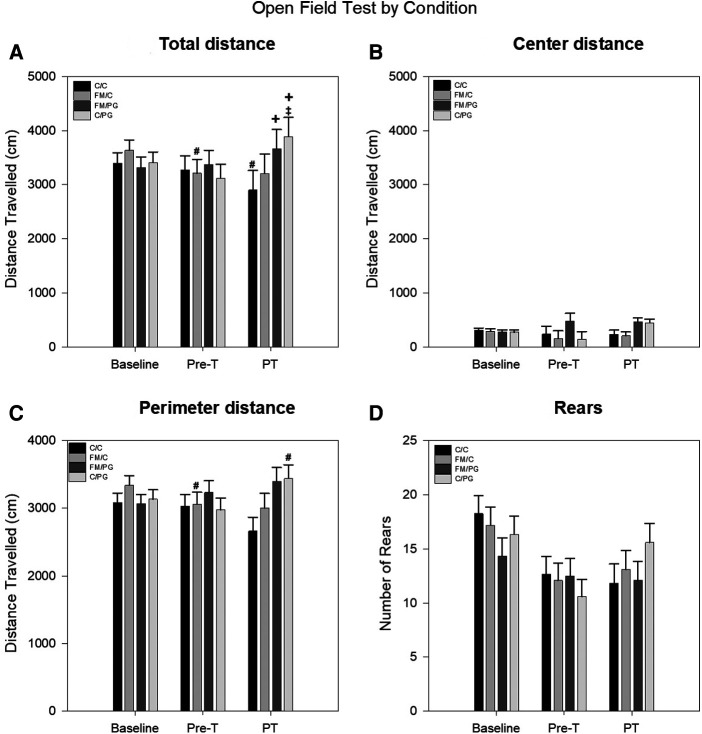
A mixed-model ANOVA was performed for each open field measure to evaluate locomotive changes between pain conditions over time. (**A**) Open field perimeter distance (cm) by pain conditions over time displayed as mean (±SEM). (**B**) Open field center distance (cm) by pain conditions over time displayed as mean (±SEM). (**C**) Total open field distance traveled (cm) by pain conditions over time displayed as mean (±SEM). (**D**) Open field rearing behavior by pain conditions over time displayed as mean (±SEM). Pre-T, Pre-treatment; PT, Post-treatment. +*p* < .05 compared to FM/C and C/C group, #*p* < .05 compared to baseline, ‡*p* < .05 compared to pre-treatment.

*Pregabalin*: Changes in locomotor activity (distance traveled along the perimeter, distance traveled in the center, total distance traveled, total number of rears) as a measure of anxiety-like behavior was analyzed using a 4 (group) × 3 (time) mixed-model ANOVA with group as the between-subjects variable and time as the within-subjects variable. The analysis of distance traveled along the perimeter indicated no significant main effect for group, *F*(3, 43) = 0.86, *p *= .472, or time, *F*(2, 86) = 0.47, *p *= .628, but there was a significant group × time interaction, *F*(6, 86) = 3.68, *p *= .003. Fisher's LSD analyses revealed that animals in the C/C group traveled less around the perimeter of the apparatus during the post-treatment assessment than at both baseline and pre-treatment. Additionally, animals in the C/PG group traveled significantly more along the perimeter of the apparatus at post-treatment assessment than compared to pre-treatment (Figure 3A). For distance traveled in the center, there were no significant differences for group, *F*(3, 44) = 2.05, *p *= .121: or time, *F*(2, 88) = 0.80, *p *= .453; and there was no significant group × time interaction, *F*(6, 88) = 1.29, *p *= .272 (Figure 3B). For total distance traveled the analysis indicated no significant main effects for group *F*(3, 44) = 0.67, *p *= .573; or time, *F*(2, 88) = 2.18, *p *= .121, However, a significant group × time interaction was identified, *F*(6, 88) = 4.21, *p *< .001. The total distance traveled for the FM/C group decreased from baseline to post-induction of the acidic saline model, while the total distance traveled by C/PG group increased following the administration of PG. It should be noted that the total distance traveled by the C/C group decreased from baseline to post-treatment, potentially indicative of a test-retest effect (Figure 3C). The evaluation of total number of rears revealed no main effect of group, *F*(3, 44) = 0.22, *p *= .883. There was a main effect of time, *F*(2, 88) = 13.23, *p *< .001, with rearing behavior decreasing following baseline measures which again is likely attributable to habituation with test-retest. The group × time interaction was not significant, *F*(6, 88) = 1.62, *p *= .152 (Figure 3D).

**Figure 4:**
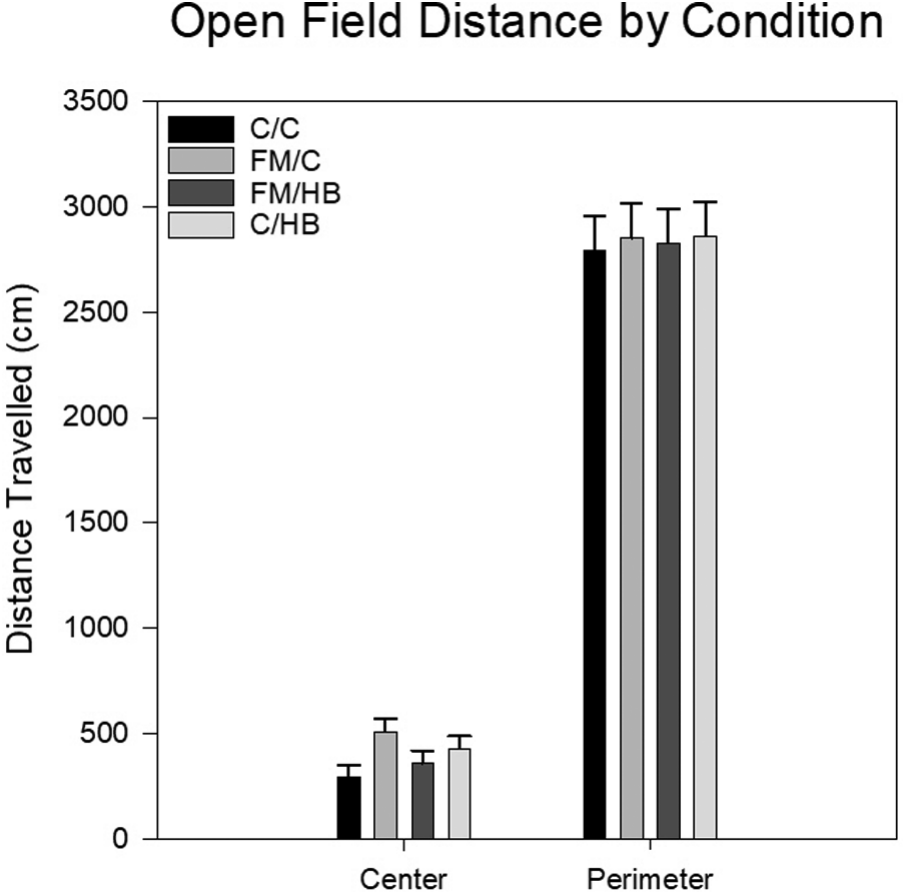
Mean distance travelled in the center and the perimeter of the open field as each analyzed with a one-way ANOVA. Center distance and perimeter distances (cm) by pain conditions displayed as mean (±SEM). No significant differences were identified.

*HBO_2_*: To analyze differences in total distance traveled within the center of the open field apparatus, a one-way analysis of variance (ANOVA) was utilized. There was no significant main effect of group, *F*(3, 44) = 2.435, *p* = .077, although it should be noted that the relationship was trending towards significance with animals in the FM/C group traveling the most distance in the center of the open field apparatus. Similarly, the analysis of distance traveled along the perimeter of the open field apparatus also revealed no significant main effects or interaction (Figure 4).

### 3.3. Place escape/avoidance paradigm

**Figure 5:**
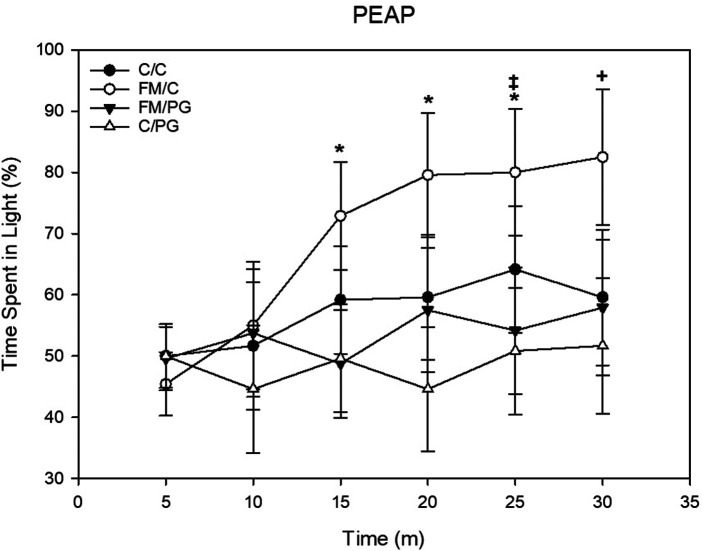
Mixed-model ANOVA of place escape/avoidance paradigm (PEAP) behavior displayed by each pain conditions’ percentage spent avoiding stimulation over time displayed as mean (±SEM). +*p* < .05 compared to all other groups, *p < .05 compared to FM/PG, ‡*p* < .05 compared to C/PG group.

*Pregabalin*: To investigate potential alterations of escape/avoidance behaviors, a 4 (group) × 6 (time) mixed-model ANOVA was used, with the twelve 5-minute time intervals of the 30-minute behavioral assessment as the within-subjects variable. There was a significant main effect of group, *F*(3, 44) = 3.28, *p *= .03, and time, *F*(5, 220) = 5.32, *p *< .001, as well as a significant group × time interaction, *F*(15, 220) = 1.90, *p *= .024. Fisher's LSD *post hoc* analyses revealed that animals in the FM/C group spent a larger percentage of time in the light side of the apparatus (i.e., demonstrated increased escape/avoidance behavior in response to the mechanical stimulus) over time compared to all other experimental conditions, indicative of increased avoidance behavior. Further, animals in the FM/PG group exhibited significantly less escape/avoidance behavior than animals who received a saline control treatment (Figure 5).

### 3.4. Sucrose preference test

**Figure 6:**
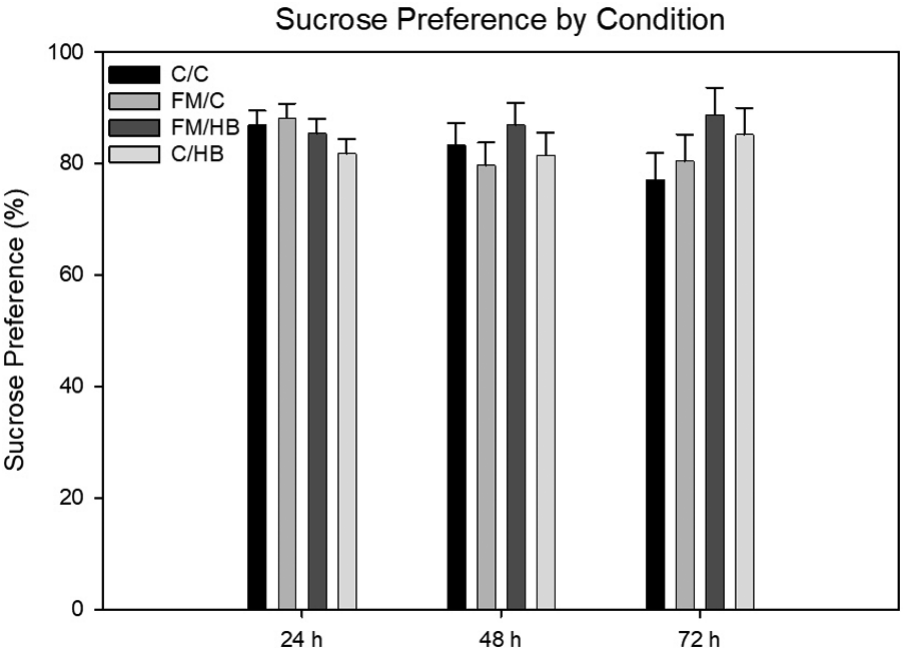
Mixed-model ANOVA of sucrose preference (%) by pain conditions over 72 h displayed as mean (±SEM). No significant differences were identified.

*HBO_2_*: To analyze differences of sucrose preference over time, we used a 4 (group) × 3 (time) mixed-model analysis of variance (ANOVA), with group as the between-subjects variable and time as the within-subjects variable. There were no significant main effects for group, *F*(1, 43) = 0.611, *p* = .611; nor time, *F*(2, 86) = 0.853, *p* = .43. The group × time interaction was also not significant, *F*(6, 86) = 0.255, *p* = .287 (Figure 6).

## 4. Discussion

The purpose of the current studies was to further investigate the efficacy of the acidic saline model of FM in replicating negative emotionality as reported in clinical manifestations of FM. Further, we sought to utilize a back-translational approach to gauge the potential efficacy of HBO_2_ as a therapeutic mechanism for the alleviation of acidic saline induced pain and its associated negative affectivity. Animals were induced into the acidic saline model and measures of anxiety and place escape/avoidance were recorded, with replication of the pharmacological profile being confirmed through the administration of pregabalin. In addition, some animals were induced into the acidic saline model, where measures of anxiety and anhedonia were recorded, with HBO_2_ being investigated as a potential therapeutic agent for alleviation of both reduced thresholds and potentially negative affect.

The results showed a reduction in mechanical thresholds among animals randomized to the FM condition, such that there were indications of hyperalgesia associated with the injections of hypertonic saline. Administration of pregabalin significantly reduced mechanical hyperalgesia. Animals randomized to the FM condition who received vehicle injections showed the most avoidance behavior during the PEAP test, implicative of negative affect associated with the FM-like pain. Of interest is that pregabalin treatment reduced the avoidance behavior to a level comparable to that of non-FM control animals. These results imply that negative affect associated with the acidic saline model can be measured using PEAP methodology (16) and is sensitive to therapeutic interventions such as pregabalin.

We had expected to see similar outcomes of anxiety-like behavior and locomotion, but there was no evidence for animals in the FM condition showing differences in anxiety-like behaviors compared to controls. Interestingly, saline control animals randomized to receive a pregabalin treatment showed an increase in locomotion at post-treatment compared to pre-treatment. This increase in locomotion provides evidence that pregabalin dosages at 30 mg/kg likely were not involved in the absence of anxiety-like behavior through the induction of lethargy or ataxia, as has been previously observed (33). However, control animals who received sham treatment showed less anxiety over each testing session, as displayed by an increase in distance traveled in the center of the apparatus. The gradual reduction of anxiety like behavior is likely due to a test–retest error and should be examined in future experiments.

Prior to HBO_2_ treatment, there was a significant difference of mechanical withdrawal thresholds between the FM condition and the saline control condition following induction of the FM model, replicating the pregabalin results and providing further validity for the acidic saline model in its ability to produce FM-like mechanical evoked pain. It was also anticipated that after each treatment session, specifically among FM animals randomized to the HBO_2_ condition, there would be an increase in paw withdrawal thresholds following each treatment session. Data indicated no significant differences in thresholds between FM animals that received HBO_2_ compared to those who received a control treatment, until 72 h following the second treatment. Then, animals in the FM condition who received HBO_2_ showed significantly lower thresholds than FM animals that received a control treatment. Though this study is the first to investigate the potential effect HBO_2_ has on the acidic saline model of FM, these results were not in line with results of the few clinical studies that exhibited HBO_2_'s efficacy in FM pain management (22–25). In fact, the results of these studies provided strong evidence within clinical populations for the use of HBO_2_ as a potential FM treatment method. Additionally, applying a back-translational approach within the current study indicated that the efficacy of HBO_2_ seen in clinical populations did not render increased thresholds among rats induced into a prominent animal model of FM pain. However, it is plausible that the treatment regimen investigated by this study did not reach the therapeutic threshold needed to produce pain alleviation. Therefore, these results from novel exploration of the back-translational application of HBO_2_ within Sluka et al.'s acidic saline model of FM (8) suggests future studies should seek to analyze variations in oxygenation and pressurization dosages, as well as the impact of more treatment schedules to potentially alleviate FM-like pain.

For measures of anhedonia, we anticipated that animals in the FM condition that received a control treatment would exhibit the greatest levels of anhedonia, as indicated by the lowest magnitude of sucrose preference. In addition, FM animals that were treated with HBO_2_ were anticipated to show sucrose preference similar to controls. Analysis of the sucrose preference data did not support these hypotheses, as no significant differences were found between groups at 24 h, 48 h, or 72 h following the second hyperbaric treatment. These results countered previous work (9, 10) indicating animals within an acidic saline model displayed significant differences in anhedonic behaviors. It should be noted, however, that the current study utilized a different, and more continuous, methodological approach compared to that of Liu and colleagues (9, 10). As a result, we believe these unique results have two potential implications: (1) the acidic saline model was not effective in producing anhedonia, or (2) the pleasure associated with sucrose consumption among rodents is more salient than the pain associated with repeated insults of acidic saline. The consistent and voluminous intake of sucrose by rats has been well-documented (34–36) and has even served to contribute to the development of rodent models of binge eating disorders and the display of addiction-like behaviors (34, 37, 38). Therefore, the natural behavioral repertoire of rats displays strong reward processing associated with sweetened solutions. It is possible that the acidic saline model was not efficacious in inducing a state of anhedonia. However, anhedonia has been documented among both clinical FM patients (39–42) and among acidic saline preclinical representations (9, 10). It is possible that the magnitude of pain produced by the model in this study was not aversive enough to compete with the associated sucrose reward and, thus, presented as an absence of anhedonic behaviors.

It was further hypothesized that animals in the FM condition that received HBO_2_ would show significantly less anxiety-like behavior within the open field paradigm indicated by spending less time along the perimeter of the apparatus. Comparably, we anticipated that animals in the FM condition that received a control treatment would show significantly more anxiety-like behavior. Contrary to our hypotheses, analyses of the distance traveled in both the center and the perimeter of the open field apparatus did not support this relationship, as there were no significant between-groups differences. Previous studies produced results indicating that FM is associated with increased anxiety-like behavior in the open field paradigm (9, 11, 12), which calls into question the complexity of the relationship between the acidic saline model and the circumstances under which the model is capable of replicating negative emotionality associated with FM.

The absence of anxiety-like behavior observed in the current studies contradicts previous studies that also utilized the open field paradigm to investigate the negative emotionality associated with the acidic saline model (9, 11, 12). Failure to show anxiolytic behavior might be attributed to errors associated with repeated measures of open field activity. However, a single between-subjects measure of open field activity produced a similar, unexpected absence of anxiety-like behavior (14, 15). The absence of a collective negative affect *and* sensory experience characteristic of FM likely produced a much less aversive experience and, thus, failed to produce changes in the behavioral repertoire of the animals that might have been observed in measures of sucrose preference or open field activity. Further investigations are necessary to identify the underlying variables that may be contributing to the variation in development of negative affect that has been previously observed.

The expected back-translational efficacy of HBO_2_ as a therapeutic agent for treatment of FM pain was derived from clinical manifestations of improved symptomology and pain thresholds associated with the treatment modality (22–25). Additionally, evidence for the use of HBO_2_ in treatment of various other presentations of preclinical pain has provided even further promise (17–21). However, the inability of this study to bridge the gap between preclinical pain research and clinical FM research might conceivably be attributed to the treatment regimen employed by this study. This study utilized two treatments of 100% oxygen at 2.0 ATA which, compared to other preclinical studies, was a rather low pressurization and number of treatment sessions (17–21). However, this treatment dosage of 2.0 ATA did prove to be beneficial for previous clinical FM samples (23–25). Overall, there is potential that variations in treatment dosages—as well as variations in the roles that pressurization and oxygenation play as independent factors in HBO_2_—may elicit more beneficial therapeutic outcomes than observed in the current study.

## 5. Conclusion

The information obtained from preclinical investigations of various pathologies and their affective dimensions serves as a crucial foundation for approaching clinical disease manifestations. In order to successfully utilize these preclinical models, understanding the full translational efficacy, including replication of pathological characteristics and cognition/affectivity, is vital. In the study of FM, a disorder with no single identified etiology or treatment, it is essential we have a complete understanding of the preclinical models used to investigate disease state variability and potential therapeutic approaches. The findings from the current study have provided controversial evidence for the ability of the acidic saline model to fully replicate the complex experience of FM by encompassing its sensory and affective elements. The degree to which we understand the relationship between commonly-observed affectivity in clinical populations versus in preclinical models directly influences further research into underlying biological and psychological mechanisms associated with less-understood pain states, such as FM. Exploring the ability of potential therapeutic modalities to alleviate negative affect associated with chronic pain serves to steer clinical treatment approaches towards a focus not just on the sensory alleviation of pain but, rather, treatment of pain as a sensory, affective, and cognitive experience. While the current study provided conflicting data on the ability of a prominent FM model to replicate its characteristic multidimensional pain experience, as well as alleviate the pain through HBO_2_ treatment, future investigations should focus on experimental factors that contribute to development of the previously observed negative affect within the acidic saline model. Additionally, further research should appraise potential alterations in HBO_2_ regimens to probe the effects on reduced thresholds produced by the acidic saline model.

## Data Availability

The raw data supporting the conclusions of this article will be made available by the authors, without undue reservation.
